# Exercise for the Teeth

**Published:** 1877-03

**Authors:** M. L. Holbrook

**Affiliations:** in the Herald of Health


*Exercise for the Teeth.*—M. L. Holbrook, M. D., in the *Herald of Health*, says:—

" There is very little doubt but the human teeth have become through successive ages of civilization more fragile and liable to decay than is either pleasant or profitable. Just how much of this is due to lack of care and cleanliness, and just how much more to food, no one can tell; but it is evident these causes are among the chief, and it would seem reasonable that man in civilization should do something to prevent it. Now in barbarous tribes of men the teeth are generally sound, and yet a tooth-brush and soap is never used, and this may be because their teeth are exercised more by hard food, and cleaned by the abundant flow of saliva which would naturally come from hard food. Man cannot, however, go back to a savage life, so he must contrive means for keeping his teeth so that they shall not decay. That one of these means is the tooth-brush and tooth-soap is certain; but is this sufficient? We think not, and believe that exercise is an additional means of toughening the teeth and making them strong. But how are they to be exercised? Surely it cannot be done on soft bread and such food as is too often found on our tables. It may be that the eating of raw wheat would give the proper exercise, and we suggest that it be tried in this way: Each day let the teeth be exercised on a teaspoonful of clean, dry, uncooked wheat. Children become fond of it, and if chewed fine it is very sweet, and promotes the rapid flow of much saliva. Unless swallowed whole it cannot do harm. It must not be supposed that any effect, however, to prevent entirely the decay of the teeth, weakened by ages of bad dietetic habits, can in one generation be effected, and so the dentist must for the present be called in to our aid.

Speaking of dentists, let me remark that a good New York dentist told me the other day that the teeth of boys brought up in New York were much worse than those of country boys.' The jaws are smaller, the teeth softer, and not always covered over with enamel. If this is so, it is a great misfortune to be born in a city ; but the dentist admitted that if the boys and girls were to chew a handful of raw wheat every day, it woutd cure the defect, so let us try it ; we exercise our horses, why not our teeth ? "

